# Breast carcinoma detection modes and death in a female population in relation to population-based mammography screening

**DOI:** 10.1186/2193-1801-3-348

**Published:** 2014-07-08

**Authors:** Tytti Sarkeala, Tapio Luostarinen, Tadeusz Dyba, Ahti Anttila

**Affiliations:** Mass Screening Registry/Finnish Cancer Registry, Unioninkatu 22, Helsinki, 00130 Finland; Finnish Cancer Registry, Unioninkatu 22, Helsinki, 00130 Finland

**Keywords:** Breast cancer, Mammography, Screening, Population, Detection

## Abstract

**Purpose:**

Associations between population-based screening, breast carcinoma detection modes and breast carcinoma death have not been studied using nationwide data at individual level. We evaluated these in Finland, where invitational age is gradually expanding from 50–59 to 50–69 years in 2008–2017. We also predicted breast carcinoma patterns in 2020 to assess the impact of changing invitational policy on breast carcinoma incidence and mortality.

**Methods:**

The data included breast carcinomas in 2000–2010 (n = 48 040), and deaths due to these carcinomas (n = 4722). We divided carcinomas into those detected before or after the screening age, and those detected at the screening age. The latter was further divided into screen-detected and interval carcinomas, and carcinomas in the non-attendees. The prediction of future patterns was based on incidence data from the ten-year period 1998–2007 preceding the period of expanding invitational age in the national programme.

**Results:**

Approximately 13% of in situ carcinomas were detected before, 29% after, and 57% at the screening age. In invasive cancers, the percentages were 16%, 42%, and 42%, respectively. At the screening age, more than half of invasive cancers were screening-detected, one quarter interval cancers, and one out of six cancers in the non-attendees. Almost 60% of breast cancer deaths were due to cancers detected after the screening age. By 2020, breast cancers detected at the screening age will increase from 42% to 65%, and breast cancers detected by screening from 23% to 38%.

**Conclusions:**

The study demonstrates a novel approach to examine associations between breast carcinoma incidence and mortality within and outside population-based screening. The results show mammography screening having a distinct role in overall breast carcinoma incidence and mortality.

**Electronic supplementary material:**

The online version of this article (doi:10.1186/2193-1801-3-348) contains supplementary material, which is available to authorized users.

## Introduction

Population-based mammography screening is a multistep process starting with identification and invitation of the target population, and continuing further to mammography test, and prospective recall examinations, cancer management and care (Vainio and Bianchini 2002; Perry et al. 2008). In the screening target population, breast cancers are detected among attendees within the screening programme, or outside the programme either among non-attendees or among attendees without findings in their previous screening episode. Additionally, a considerable amount of breast carcinomas are detected in women who not yet have reached, or already have passed, the screening age.

Monitoring of the European population-based mammography screening has shown significant variation between programmes and centres in invitational coverage, attendance, and sensitivity and specificity of screening (Sarkeala et al. 2004; Giordano et al. 2012; Hofvind et al. 2012). Relationships between the screening process (recall rate), the screening performance (sensitivity), and the mortality reduction due to screening have also been shown to be non-straightforward (Sarkeala et al. 2006). The above imply delicate balance between benefits and harms in the European population-based mammography screening.

Variation in the screening performance (Sarkeala et al. 2006; Törnberg et al. 2010) – as well as in the coverage of screening – affects the ratio of breast carcinomas detected within and outside the organised screening. Furthermore, a considerable percentage of breast carcinomas detected outside screening may be asymptomatic with a prognostic profile comparable to that of the screen-detected carcinomas (Sarkeala et al. 2004; 2006; Hoff et al. 2012). Hence it is important to assess the detection of breast carcinomas in the whole female population.

Albeit recommended by the European Network of Cancer Registries (European Network for Cancer Registries 2014), only few European countries have so far assessed the impact of population-based mammography screening on the distribution of all breast carcinomas. This would, however, help to assess associations between breast carcinoma diagnostics and treatment, and to clarify impacts of population-based mammography screening on breast carcinoma incidence and mortality.

The aim of the current study is to examine detection modes of incident breast carcinomas and breast carcinoma deaths in relation to invitation and participation to population-based mammography screening. The study uses all breast carcinomas among Finnish women in 2000–2010, and deaths due to these carcinomas. Additionally, the study analyses distribution and trend of breast carcinomas in areas practicing various invitational policies, and predicts breast cancer patterns in 2020, when the national mammography programme in Finland will cover all women aged 50–69 years.

## Material and methods

In Finland, population-based mammography screening was started gradually in 1986–1987. The screening programme was fully implemented in the beginning of 1992 for all women aged 50–59 years. Detailed description of the programme has been given earlier (Sarkeala et al. 2004; 2006). Based on the Government Degree on Screenings from 2007, the invitational age of the screening programme is gradually expanding from 50–59 years to 50–69 years (Government Decree on Screenings (1339/2006)). Women born in 1947 are the first to receive a regular, biennial invitation until the age of 69 years. In the study period 2000–2010, the Finnish municipalities thus have invited all women aged 50–59 years regularly, and women aged 60–69 years by an annually expanding protocol to population-based mammography screening. The city of Turku, however, has carried out an exceptional invitational protocol throughout 2000–2010 by inviting women aged 50–69 years regularly and women aged 40–49 and 70–74 years irregularly to mammography screening.

Since the start of the national programme, the data on mammography screening have been registered centrally to the Mass Screening Registry. The coverage of registration has increased steadily from 70% to more than 98% from the early 1990’s (Mass Screening Statistics in Finland 2014). The clinical and pathological data on all breast carcinomas in Finland have been recorded by the Finnish Cancer Registry since 1953.

We assessed the detection of all breast carcinomas diagnosed in Finland in the period 2000–2010 (n = 48 040) by linking information on breast carcinomas and on screening invitations and findings at an individual level. To assess relationships between breast cancer detection and breast cancer death, we also linked the incident breast carcinoma cases with deaths from breast cancer in 2000–2010. The linkage was done using a unique personal identifier (social security number) as a key. Within this linkage procedure, we divided breast carcinomas into three categories: carcinomas detected before or after the screening age (before the first and after the last invitation to screening) and carcinomas detected at the screening age. The categorization was based on novel recommendations on the categories of detection, developed in an expert work group in a cancer-registry-driven Eurocourse project (EUROCOURSE: EUROpe against Cancer 2014). A similar assessment has earlier been reported for cervical carcinomas in Finland (Lönnberg et al. 2013).

Carcinomas detected at the screening age were further divided into screen-detected carcinomas, interval carcinomas, carcinomas among the non-attendees, and carcinomas among women, whose data on screening invitations and results had not been sent to the Mass Screening Registry (non-registered carcinomas). Screen-detected carcinomas were carcinomas diagnosed among the screening attendees within six months after the previous population-based mammography test with a positive result both from the test and the recall examinations. Interval carcinomas were carcinomas diagnosed among the screening attendees in between two subsequent invitations with a) a negative result from the previous test, b) a positive result from the previous test and a negative result from the previous recall examinations, or c) a positive result from the previous test and the recall examinations with a date of diagnosis six months after the date of mammography (women having both screen-detected and interval breast cancers).

We report the incident breast carcinoma categories by behaviour (in situ carcinoma of the breast; invasive carcinoma i.e. breast cancer), morphology (ductal, lobular, others), and stage (local, metastasized, unknown). The ductal carcinomas are those with ICD-O-3-codes M8500-07, and lobular carcinomas those with an ICD-O3-code M8520. The metastasized breast cancers are those metastasized to regional lymph nodes only (regional metastasis) or further and/or to adjacent tissues (distant metastasis).

To examine the proportions of screen-detected and interval breast cancers and breast cancers among the non-attendees out of all breast cancers in the screening target population in 2000–2010, we applied Poisson regression. Period wise (2000–2002 (reference period), 2003–2006, and 2007–2010) estimates of incidence rate ratio (IRR) were adjusted for categorical age (50–54, 55–59, 60–64, and 65–69 years).

Based on the varying invitational policies between the rest of Finland and the Turku area in 2000–2010, and incidence prediction by an additive Poisson model (Dyba and Hakulinen 1994), we estimated the distribution of invasive breast cancers in Finland in 2020, when all women aged 50–69 years are expected to get an invitation to mammography screening in every two years (Government Decree on Screenings (1339/2006)). The Poisson model allowed for different age-specific changes of incidence rates on an additive scale over time (Dyba and Hakulinen 2000). The prediction was based on breast cancer incidence in ages 30–89 years in the ten-year period 1998–2007 preceding the expansion period of the invitational age in the national screening programme. The predicted, age-specific numbers of breast cancers in ages 30–89 years in 2020 were divided into breast carcinoma categories using information on breast cancer detection from 2000–2010 from Turku area for ages 60–79 years, and corresponding information from the rest of Finland for ages less than 60 and more than 79 years.

## Results

**Figure 1:**
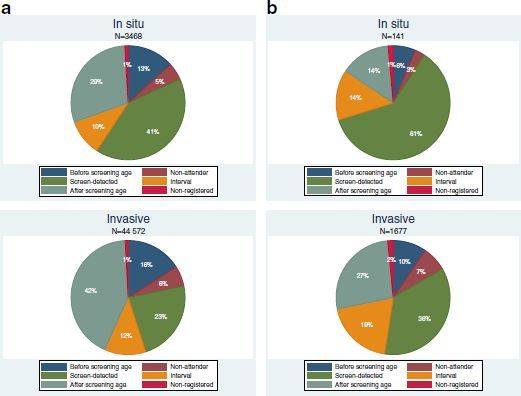
**Breast carcinoma detection modes in 2000-2010. (a)** In situ breast carcinomas and invasive breast cancers in relation to invitation to screening in the whole country. **(b)** In situ breast carcinomas and invasive breast cancers in relation to invitation to screening in the Turku area.

A total of 3468 in situ carcinomas and 44 572 invasive breast cancers were diagnosed in Finland in 2000–2010 (Figure 1a). Approximately 13% of in situ carcinomas were detected before the first, 29% after the last invitation to screening, and 57% at the screening age. In invasive cancers, the corresponding percentages were 16%, 42%, and 42%. At the screening age, more 55% of the invasive breast cancers were detected by screening, 28% as interval cancers, and 14% among the non-attendees. In Turku area, where women aged 50–69 had been invited regularly to mammography screening throughout 2000–2010, most invasive breast cancers (64%) were detected at the screening age (Figure 1b).

At the screening age, most breast carcinomas were detected in ages 50–69 years (Table 1). Few cases in age groups less than 50 and over 69 years were detected in Turku city, where the age groups 40–49 and 70–74 years had been invited to population-based screening irregularly in 2000–2007. Most interval cancers were due to negative result from the mammography test.

**Table 1:** **Breast carcinomas by behaviour and age in relation to invitation to screening in the screening target population in 2000–2010**

	In situ							
	< 50 yrs		50-69 yrs		>69 yrs		Total	
	N	(%)	N	(%)	N	(%)	N	(%)
**ALL**	**19**	**(100)**	**1948**	**(100)**	**18**	**(100)**	**1 985**	**(100)**
Non-attendee	2	(11)	165	(9)	2	(11)	169	(9)
Screen-detected	9	(47)	1411	(72)	7	(39)	1 427	(72)
Interval	2	(11)	346	(18)	8	(44)	356	(18)
test-	2	(11)	252	(13)	6	(33)	260	(13)
test+, recall-	-	(−)	61	(3)	2	(11)	63	(3)
test+, recall+, dg>6 mths	-	(−)	33	(2)	-	(−)	33	(2)
Non-registered	6	(32)	26	(1)	1	(6)	33	(2)
	**Invasive**							
	**< 50 yrs**		**50-69 yrs**		**>69 yrs**		**Total**	
	**N**	**(%)**	**N**	**(%)**	**N**	**(%)**	**N**	**(%)**
**ALL**	**227**	**(100)**	**18 139**	**(100)**	**198**	**(100)**	**18 564**	**(100)**
Non-attendee	31	(14)	2 512	(14)	31	(16)	2 574	(14)
Screen-detected	70	(31)	10 242	(56)	70	(35)	10 382	(56)
Interval	46	(21)	5 093	(28)	95	(48)	5 234	(28)
test-	42	(19)	4 529	(25)	80	(40)	4 651	(25)
test+, recall-	4	(2)	479	(3)	9	(5)	492	(3)
test+, recall+, dg>6 mths	-	(−)	85	(<1)	6	(3)	91	(<1)
Non-registered	80	(35)	292	(2)	2	(1)	374	(2)

Localized (63%) and regionally metastasized (29%) were the most common stages of screen-detected cancers (Table 2). In breast cancers among the non-attendees, the corresponding proportions were 43% and 36%, and in the interval breast cancers 49% and 38%, respectively, showing that stages with a favourable prognosis were largely diagnosed in the screening target population also outside the screening programme. The stage distribution of breast cancers detected before the screening age resembled to that of the non-attendees (with less progressed metastasized tumours), and the distribution of breast cancers detected after the screening age to that of the interval breast cancers (Table 2). The morphological distribution of invasive breast cancers was similar within and outside the programme.

**Table 2:** **Invasive breast cancers by stage and morphology in relation to invitation to screening in 2000–2010**

	Before screening age		At screening age								After screening age		Total	
			Non-attendees		Screen-detected		Interval		Non-registered					
	N	(%)	N	(%)	N	(%)	N	(%)	N	(%)	N	(%)	N	(%)
**ALL**	**7 129**	**(100)**	**2 574**	**(100)**	**10 382**	**(100)**	**5 234**	**(100)**	**374**	**(100)**	**18 879**	**(100)**	**44 752**	**(100)**
Local	3056	(42)	1 110	(43)	6 495	(63)	2 563	(49)	195	(52)	9 089	(48)	22 508	(50)
Metastasis	3698	(51)	1 272	(50)	3 290	(31)	2 392	(46)	157	(42)	7 881	(42)	18 690	
regional	3 140	(44)	938	(36)	3024	(29)	2 015	(38)	138	(37)	5 695	(30)	14 950	(42)
distant	427	(6)	284	(11)	144	(1)	280	(5)	17	(5)	1 740	(9)	2 892	(6)
not specified	131	(1)	50	(3)	122	(1)	97	(3)	2	(<1)	446	(2)	848	(2)
Unknown	375	(7)	192	(7)	597	(6)	279	(5)	22	(6)	1 909	(10)	3 374	(8)
Ductal	5 586	(78)	1 866	(72)	7 976	(77)	3 767	(72)	287	(77)	13 164	(70)	32 646	(73)
Lobular	1 010	(14)	426	(17)	1 672	(16)	1 037	(20)	66	(18)	3 395	(18)	7 606	(17)
Others	533	(8)	282	(11)	734	(7)	430	(8)	21	(5)	2 320	(12)	4 320	(10)

**Figure 2:**
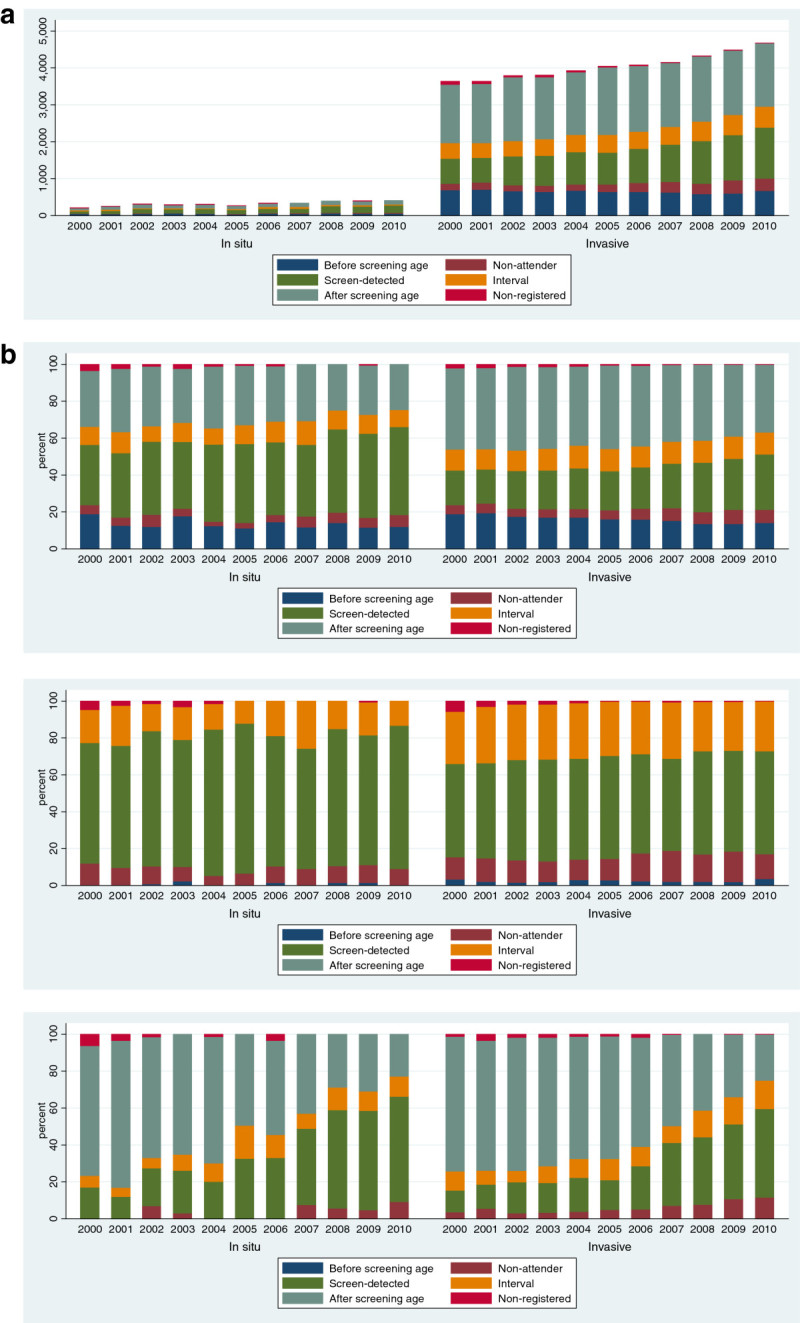
**Breast carcinoma detection modes over 2000-2010. (a)** Absolute numbers of in situ breast carcinomas and invasive breast cancers in relation to invitation to screening, all ages. **(b)** Proportion of in situ breast carcinomas and invasive breast cancers in relation to invitation to screening; all ages, ages 50-59, and ages 60-69 years.

The absolute numbers of in situ carcinomas and invasive breast cancers increased clearly from 2000 to 2010 (Figure 2a). At the screening age, the proportion of interval breast cancers was 13% lower in the expansion period 2007–2010 compared to the period 2000–2002 (IRR 0.87, 95% confidence interval (CI) 0.81-0.93%) (Figure 2b, Table 3). This was, however, mainly attributable to the ages 60–69 years (Table 3). The increase in the proportion of breast cancers among the non-attendees from 2000–2002 to 2007–2010 was more pronounced, 18% (IRR 1.18, 95% CI 1.07-1.31) than that of the screen-detected breast cancers, 11% (IRR 1.11, 95% CI 1.05-1.16) (Table 3). The increase in non-attendees was due to ages 50–59 years (Table 3), which were covered by the screening programme already before the year 2000.

**Table 3:** **Development of breast cancers among the non-attendees, screen-detected breast cancers, and interval breast cancers from 2000–2002 to 2007–2010 (expansion period) by incidence rate ratios (IRR) with 95% confidence intervals (95% CI) in ages 50–59, 60–69, and 50–69 years**

50-59 years						
	Non-attendee		Screen-detected		Interval	
	IRR	95% CI	IRR	95% CI	IRR	95% CI
**Period**						
**2000-2002**	**1**		**1**		**1**	
**2003-2006**	0.98	0.87-1.10	1.07	1.01-1.13	0.97	0.90-1.05
**2007-2010**	1.23	1.09-1.38	1.05	0.99-1.11	0.92	0.85-0.99
**60-69 years**						
	**Non-attendee**		**Screen-detected**		**Interval**	
	**IRR**	**95% CI**	**IRR**	**95% CI**	**IRR**	**95% CI**
**Period**						
**2000-2002**	**1**		**1**		**1**	
**2003-2006**	0.83	0.65-1.06	1.07	0.95-1.21	1.09	0.93-1.29
**2007-2010**	1.04	0.84-1.29	1.26	1.13-1.41	0.77	0.66-0.89
**50-69 years**						
	**Non-attendee**		**Screen-detected**		**Interval**	
	**IRR**	**95% CI**	**IRR**	**95% CI**	**IRR**	**95% CI**
**Period**						
**2000-2002**	**1**		**1**		**1**	
**2003-2006**	0.95	0.85-1.06	1.06	1.01-1.12	0.99	0.93-1.07
**2007-2010**	1.18	1.07-1.31	1.11	1.05-1.16	0.87	0.81-0.93

Compared to the study period 2000–2010, the proportion of breast cancers detected at the screening age in 2020 is, according to the prediction, going to increase from 42% to 65% (Table 4). Of these, the proportion of screen-detected and interval cancers will increase, and the proportion of breast cancers among the non-attendees will remain more or less at the same level.

**Table 4:** **Invasive breast cancers by behaviour in relation to invitation to screening in the Finnish female population in the study period 2000–2010 and the corresponding predicted values with 95% confidence intervals (CI) in 2020**

	2000-2010		2020		
	N	(%)	N	95% CI	(%)
ALL	44 572	(100)	5 454		(100)
Before screening age	7 129	(16)	541	(496–589)	(10)
At screening age	18 564	(42)	3 545	(3 429–3 662)	(65)
non-attendee	2 948	(7)	410	(371–451)	(8)
screen-detected	10 291	(23)	2 076	(1 988–2 166)	(38)
interval	5 325	(12)	1 059	(996–1 124)	(19)
After screening age	18 879	(42)	1 368	(1 296–1 441)	(25)

**Figure 3:**
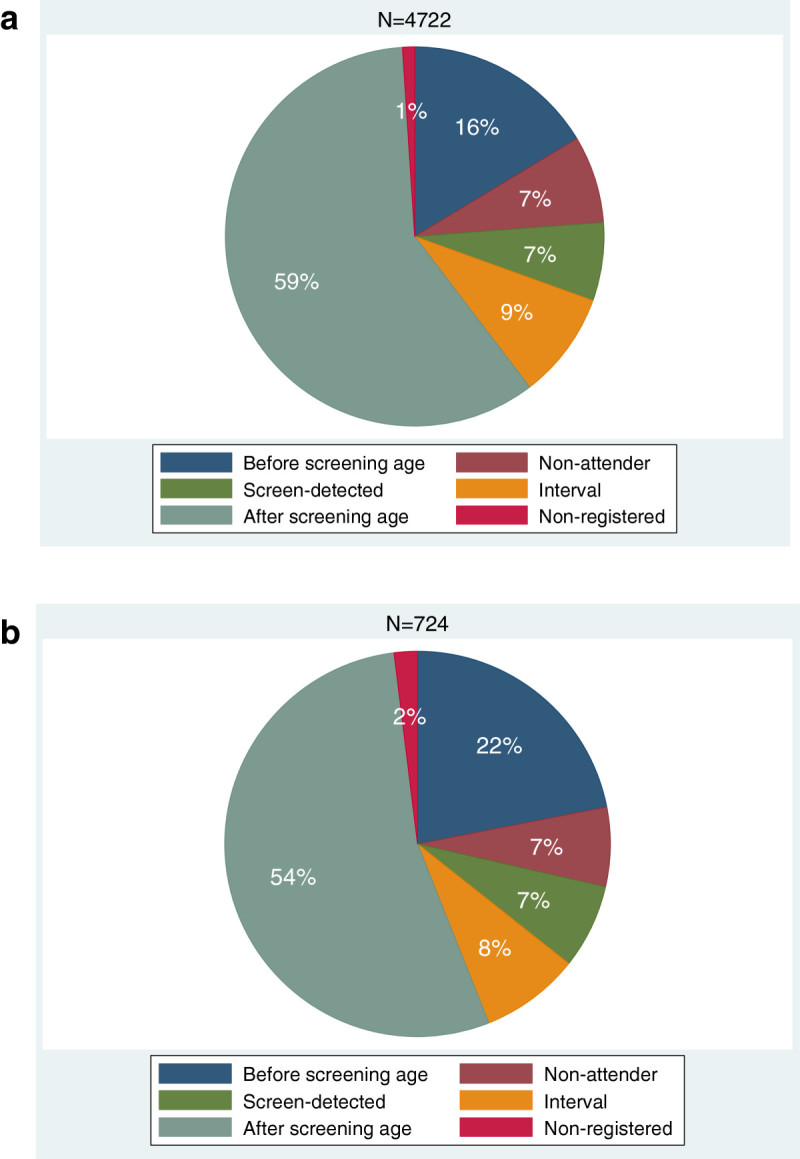
**Death from breast cancer in relation to breast carcinoma detection modes. (a)** Both diagnosis and death in 2000-2010. **(b)** Diagnosis in 2000, death in 2000-2010.

When assessing breast cancer deaths in 2000–2010, those resulting from breast cancers detected throughout 2000–2010 (potential follow-up time 1–11 years, n = 4722) were mostly, 59%, due to breast cancers detected after the screening age (Figure 3). The same phenomenon was also seen in breast cancers detected during the year 2000 only (potential follow-up time 11 years, n = 724). Only 7% of breast cancer deaths were due to breast cancers detected by screening. In interval cancers and cancer among the non-attendants, the corresponding proportions were 9% and 7%.

## Discussion

We examined all breast carcinomas in Finland in relation to invitation, attendance and findings in the population-based mammography screening, and deaths due to these carcinomas. We also assessed the impact of the expansion of invitational age on the detection of breast carcinomas in the future. To our knowledge, the study is the first to evaluate associations between population-based screening, breast carcinoma detection and breast carcinoma death in a nationwide female population.

According to our prediction, more than 60% of invasive breast cancers will be diagnosed at the screening target age and approximately 40%, respectively, directly by screening when the whole female target population aged 50 to 69 years will be invited to screening in Finland in 2020. Since breast cancers detected after the last screening round will also be affected by the preceding period of population-based screening (Day et al. 1989), screening will be involved in the characteristics of most incident breast carcinomas in 2020. Moreover, most deaths due to breast carcinoma in the study period were due to breast cancers detected after the screening age suggesting that the expanding mammography programme will also have a considerable impact on the future breast cancer mortality.

In the current data, as much as 40% of in situ breast carcinomas were detected before or after the screening age. Additionally, 10% of them were interval cancers, and 5% were detected in the non-attendees of screening. All these refer to high opportunistic activity and should be studied further. Unfortunately, the opportunistic mammography screening is not registered in Finland, and the level of this phenomenon, therefore, is difficult to measure. Other European countries have also reported increase in opportunistic activity due to population-based screening (Boncz et al. 2008).

The proportion of interval cancers in the whole screening target population was 28%, and among the screening attendees 33%. In our previous study from the 1990s and early 2000s, the proportion of interval cancers among the screening attendees measured by the detection method was 35%, and there was an increasing trend in the interval cancer proportion (Sarkeala et al. 2006). According to the current results, the reported increase in the early 2000’s seems to have turned to a decrease after the year 2007 when the expansion of invitational age in the Finnish mammography programme started. In women aged 60–69 years, the interval cancer occurrence has consistently been reported to be smaller than in the younger target population (Sarkeala et al. 2006; Törnberg et al. 2010). Nonetheless, also trends in the access to mammograms and/or other diagnostic activities outside screening may have affected the phenomenon.

The increase in the proportion of breast cancers among the non-participants – which was evident in ages 50–59 years – raises concern on the development of attendance rate in the Finnish mammography programme. According to the national statistics, overall attendance to screening has decreased during the 2000s from approximately 87 to 85 percent (Government Decree on Screenings (1339/2006)). Compared to other European countries, the overall rate in Finland is, however, still among the highest (Giordano et al. 2012).

Approximately 40% of breast carcinoma cases and more than half of breast cancer deaths were due to breast cancers diagnosed after the screening age. This was similarly evident in breast cancers detected in the study period 2000–2010 (potential follow-up time varying between 1 to 11 years) and in breast cancers detected solely during the year 2000 (potential follow-up time 11 years). In both settings, there were very few deaths due to in situ carcinoma. Also the proportion of deaths due to screen-detected cancers was small compared to the proportion they represented in the distribution of cancers (7% vs. 23%) whereas the proportion of deaths due to breast cancers among non-attendants (7% vs. 6%) and due to interval breast cancers (9% vs. 12%) was more or less similar to that of cancer distribution.

According to the prediction, the proportion of breast cancers detected at the screening age will increase from around 40% to more than 60% in Finland in 2020. When assessing this information in line with the information on breast cancer deaths, two issues arise. First, effectiveness of breast cancer screening will probably improve since older women will be involved in the programme - and screening has been shown to be more effective among women aged 60–69 years than at younger ages (Anttila et al. 2008; Sarkeala et al. 2008a; Sarkeala et al. 2008b; Otto et al. 2012; Njor et al. 2012). Second, expansion of invitational age to 70–74 years should be taken into consideration in future scenarios of the national programme. Women aged 70–74 years have been invited to population-based screening in the Netherlands since the early 1998, and the results on performance and mortality reduction have been encouraging (Broeders et al. 2002; Fracheboud et al. 2006). In Finland, women aged 70–74 years have previously been invited to population-based screening in the Turku city, where the most prominent reduction in breast cancer mortality was observed in the oldest age groups (Parvinen et al. 2006).

Current data will be utilized further to investigate associations between breast carcinoma diagnostics, therapeutic activities, and breast carcinoma death within and outside the screening target population (Lehtimäki et al. 2011; Haukka et al. 2011). Documentation outside the target population may e.g. provide information on reasons behind the reduction in breast cancer mortality in women aged 40–49 years (Anttila and Martin-Moreno 2013), which recently has been reported from several European countries. In the screening target population, the data can be applied to examine over-diagnosis and other disadvantages of screening (Hofvind et al. 2012; Puliti et al. 2012; Welch and Black 2010; Esserman et al. 2013; Bleyer and Welch 2012). A 1-10% over-diagnosis in a woman’s lifetime has been reported due to screening in ages 50–69 years (Puliti et al. 2012). If the excess of 10% in breast cancer incidence was attributed only to screening, approximately 25% (10/38*100) of screen-detected breast cancers would represent over-diagnosis in the current data (in 2020). However, stages with a favourable prognosis were largely diagnosed also outside the organised screening in the current data. This refers that over-diagnosis may be attributed also to interval breast cancers and breast cancers detected from the non-attendees.

To conclude, screen-detected in situ and invasive carcinomas represented the largest category of breast carcinomas detected at the screening age. However, stages with favourable prognosis were largely diagnosed also outside the screening programme referring that over-diagnosis may not solely be attributed to the programme. Most breast cancer deaths were due to invasive breast cancers detected after the screening age. Due to expanding invitational age, the proportion of breast cancers detected at the screening age and by screening will substantially increase by 2020. These imply that organised screening will have a distinct impact on overall breast cancer incidence and mortality in the future.

Our study demonstrates a novel approach for cancer registries to examine associations between breast cancer detection and deaths due to breast cancer. The approach requires, however, comprehensive data on screening and cancer deaths from nationwide, high quality registers with a 100% coverage and may therefore be unattainable for some European countries. In countries with appropriate databases, monitoring and evaluation of the impact of population-based screening on the overall cancer burden can be carried out on a regular basis.

## Authors’ original submitted files for images

Below are the links to the authors’ original submitted files for images. Authors’ original file for figure 1 Authors’ original file for figure 2 Authors’ original file for figure 3 Authors’ original file for figure 4 Authors’ original file for figure 5 Authors’ original file for figure 6 Authors’ original file for figure 7 Authors’ original file for figure 8 Authors’ original file for figure 9 Authors’ original file for figure 10 Authors’ original file for figure 11 Authors’ original file for figure 12 Authors’ original file for figure 13 Authors’ original file for figure 14 Authors’ original file for figure 15
