# Supplementary material for: Breast carcinoma detection modes and death in a female population in relation to population-based mammography screening
Source: Springerplus. 2014 Jul 8;3:348. doi: 10.1186/2193-1801-3-348 (PMC4796436; doi:10.1186/2193-1801-3-348)
Supplement: Supplementary file 1 — Authors’ original file for figure 1 [file 40064_2014_1477_MOESM1_ESM.pdf]

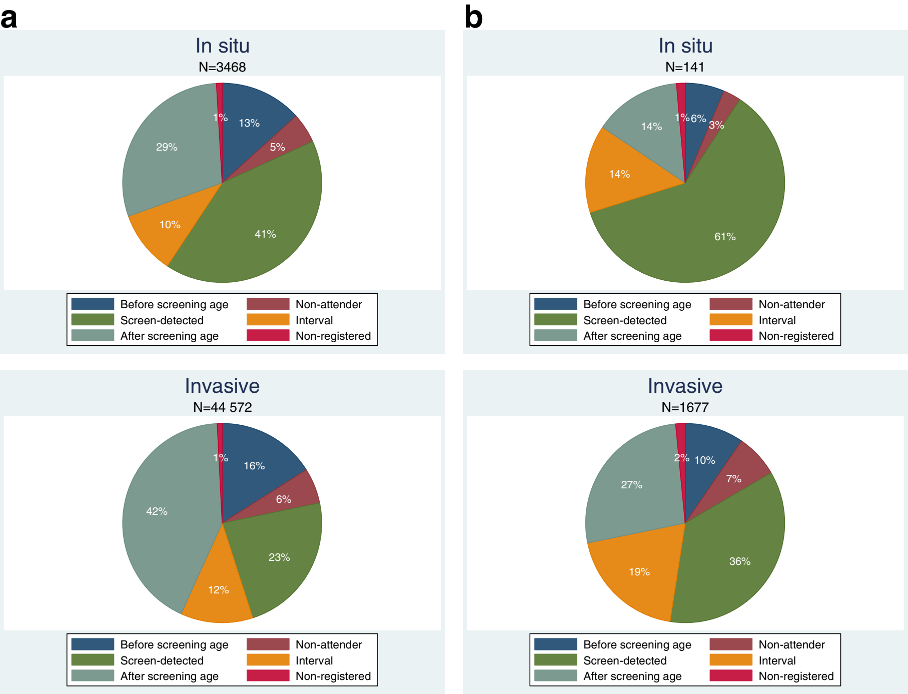

a
In situ
N=3468
1%
13%
5%
29%
10%
41%
Before screening age
Non-attender
Screen-detected
Interval
After screening age
Non-registered
b
In situ
N=141
1%
6%
3%
14%
14%
61%
Before screening age
Non-attender
Screen-detected
Interval
After screening age
Non-registered
Invasive
N=44 572
1%
16%
6%
42%
23%
12%
Before screening age
Non-attender
Screen-detected
Interval
After screening age
Non-registered
Invasive
N=1677
2%
10%
7%
27%
36%
19%
Before screening age
Non-attender
Screen-detected
Interval
After screening age
Non-registered
