# Supplementary figures and images for: Breast carcinoma detection modes and death in a female population in relation to population-based mammography screening
Source: Springerplus. 2014 Jul 8;3:348. doi: 10.1186/2193-1801-3-348 (PMC4796436; doi:10.1186/2193-1801-3-348)

**a**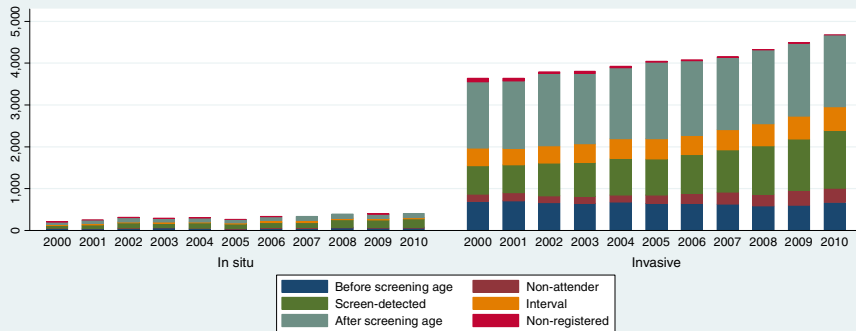**b**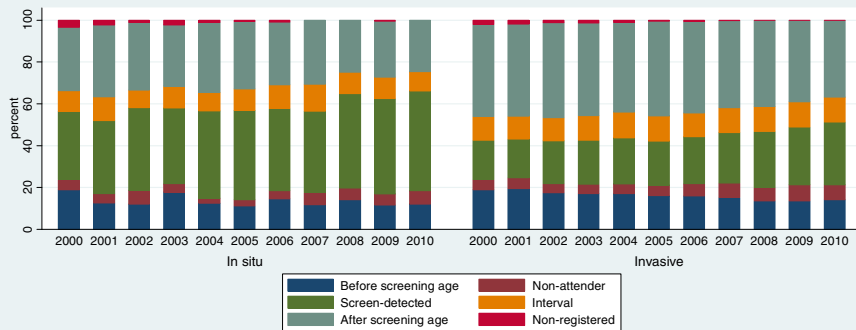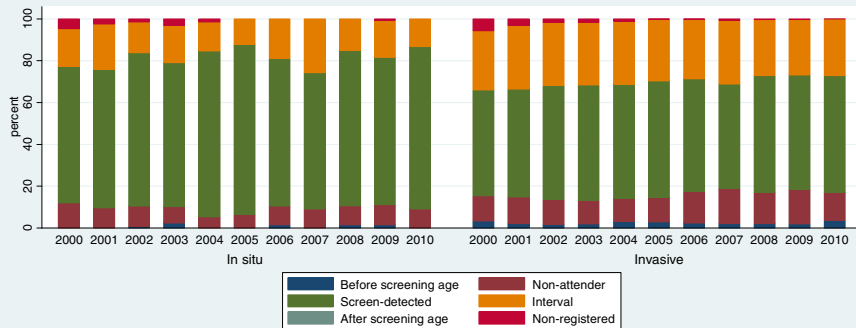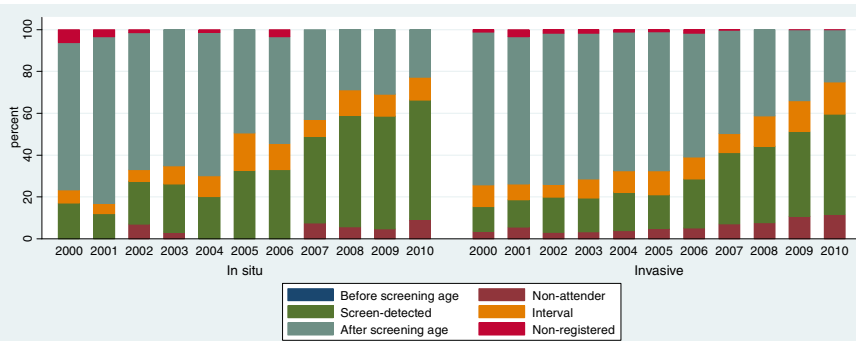

Supplement: Supplementary file 2 — Authors’ original file for figure 2 [file 40064_2014_1477_MOESM2_ESM.pdf]

**a**

N=4722

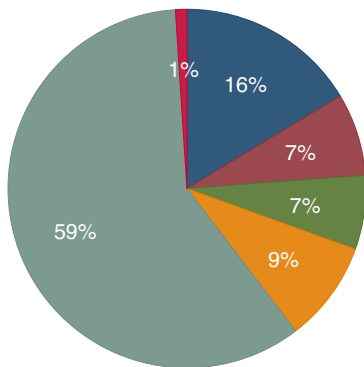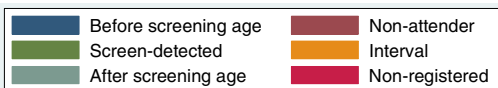**b**

N=724

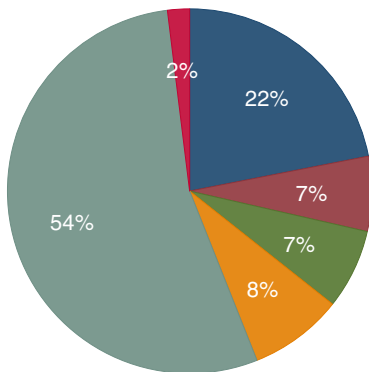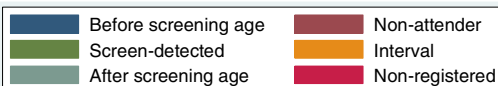

Supplement: Supplementary file 3 — Authors’ original file for figure 3 [file 40064_2014_1477_MOESM3_ESM.pdf]

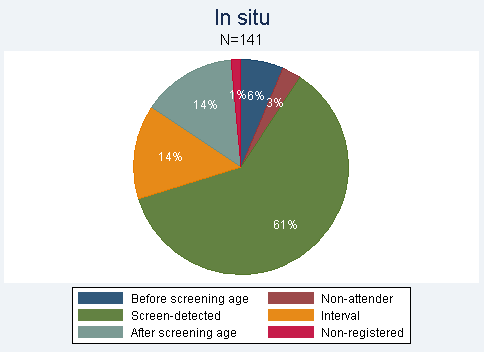

Supplement: Supplementary file 4 — Authors’ original file for figure 4 [file 40064_2014_1477_MOESM4_ESM.tiff]

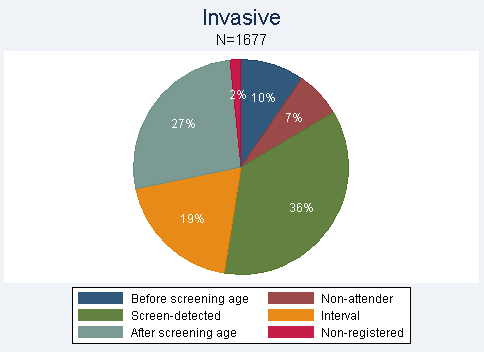

Supplement: Supplementary file 5 — Authors’ original file for figure 5 [file 40064_2014_1477_MOESM5_ESM.tiff]

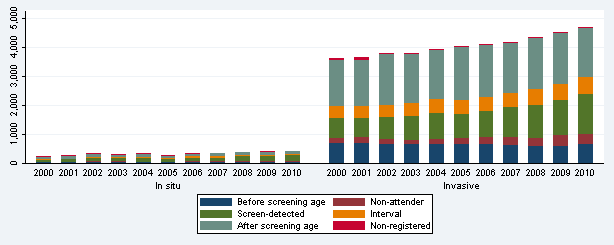

Supplement: Supplementary file 7 — Authors’ original file for figure 7 [file 40064_2014_1477_MOESM7_ESM.tiff]

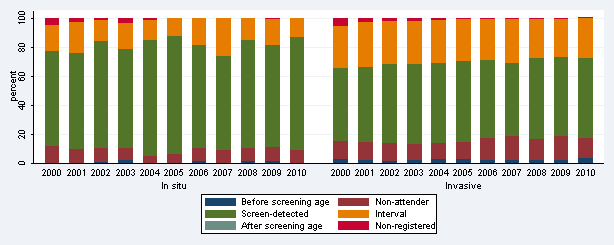

Supplement: Supplementary file 10 — Authors’ original file for figure 10 [file 40064_2014_1477_MOESM10_ESM.tiff]

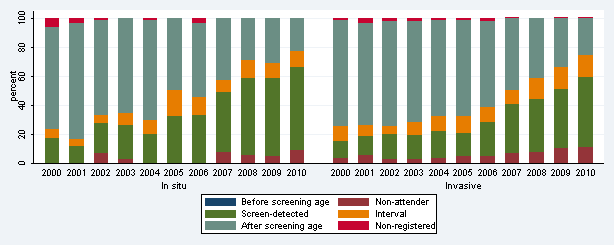

Supplement: Supplementary file 11 — Authors’ original file for figure 11 [file 40064_2014_1477_MOESM11_ESM.tiff]

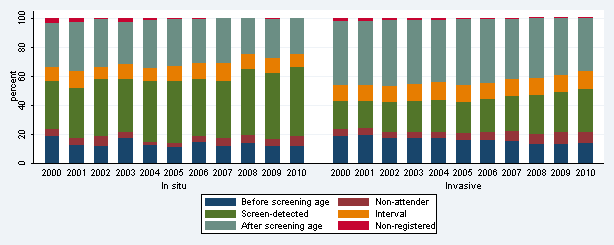

Supplement: Supplementary file 12 — Authors’ original file for figure 12 [file 40064_2014_1477_MOESM12_ESM.tiff]

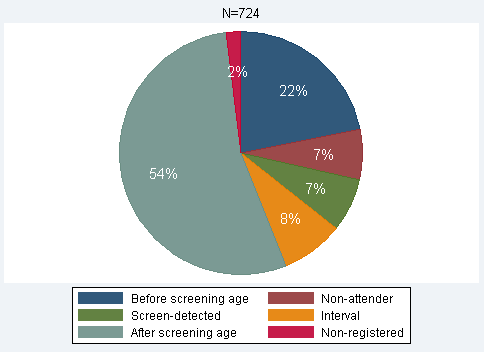

Supplement: Supplementary file 13 — Authors’ original file for figure 13 [file 40064_2014_1477_MOESM13_ESM.tiff]

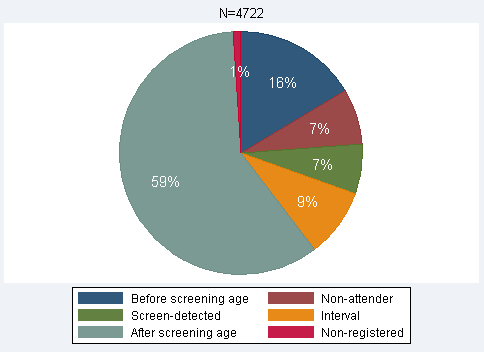

Supplement: Supplementary file 14 — Authors’ original file for figure 14 [file 40064_2014_1477_MOESM14_ESM.tiff]
