# Supplementary material for: Breast carcinoma detection modes and death in a female population in relation to population-based mammography screening
Source: Springerplus. 2014 Jul 8;3:348. doi: 10.1186/2193-1801-3-348 (PMC4796436; doi:10.1186/2193-1801-3-348)
Supplement: Supplementary file 8 — Authors’ original file for figure 8 [file 40064_2014_1477_MOESM8_ESM.docx]

**Figure 2a.** Absolute numbers of in situ breast carcinomas and invasive breast cancers in relation to invitation to screening over 2000-2010, all ages.
