# Supplementary material for: Breast carcinoma detection modes and death in a female population in relation to population-based mammography screening
Source: Springerplus. 2014 Jul 8;3:348. doi: 10.1186/2193-1801-3-348 (PMC4796436; doi:10.1186/2193-1801-3-348)
Supplement: Supplementary file 15 — Authors’ original file for figure 15 [file 40064_2014_1477_MOESM15_ESM.docx]

**Figure 3.** Breast carcinoma categories in relation to death from breast cancer.

Both diagnosis and death in 2000-2010 (N=4722)

Diagnosis in 2000, death in 2000-2010 (N=724)
